# Visual outcome in hypermetropic eye post-refractive surgery: retrospective cohort study

**DOI:** 10.1097/MS9.0000000000004165

**Published:** 2025-10-21

**Authors:** Ashjan Bamahfouz, Elham Alharbi, Anas Alqurashi, Osama Qasim, Jumanah Alsairafi, Rola Alsulami, Bedoor Aldaadi, Reham Alsaud, Ahmed Basheikh

**Affiliations:** a Anterior Segment Consultant, Department of Ophthalmology, Umm Al-Qura University, Makkah, Saudi Arabia; b Ophthalmology Department, Saudi Commission of Health Specialists, Makkah, Saudi Arabia; c Faculty of Medicine, Umm Al-Qura University, Makkah, Saudi Arabia; d Department of Ophthalmology, Faculty of Medicine, King Abdulaziz University, Jeddah, Saudi Arabia

**Keywords:** cohort study, hypermetropia, LASIK, ophthalmology, PRK, refractive surgery, Saudi Arabia

## Abstract

**Background:**

Correction of hypermetropia with laser refractive surgery is considered difficult and challenging compared to myopia, with few studies to make evidence-based decisions. This study assessed visual outcomes and complications in hypermetropic patients after refractive surgery.

**Methods:**

This retrospective cohort study included 105 patients with hypermetropia who underwent either laser *in situ* keratomileusis or photo-refractive keratectomy at a specialized eye center in Jeddah, Kingdom of Saudi Arabia. Multiple preoperative factors were evaluated and compared to 1-month postoperative factors.

**Results:**

The patient cohort had a mean age of 38.1 ± 12.0 years, 67 (63.8%) of the patients were female, and laser *in situ* keratomileusis was performed on 84 (80%) cases. Preoperative central corneal thickness in the right and left eyes was 559 ± 37 and 560 ± 37 (*P* = 0.316), respectively. The preoperative and 1-month postoperative uncorrected visual acuity was statistically significantly improved in the right eye, from 0.62 ± 0.32 to 0.91 ± 0.27 (*P* = 0.025), and in the left eye, from 0.62 ± 0.31 to 0.89 ± 0.27 (*P* = 0.043). The preoperative and postoperative spherical equivalent showed a statistically significant reduction in the right eye from 2.47 ± 1.24 to 0.19 ± 0.96 (*P* = 0.001) and in the left eye from 2.64 ± 1.73 to −0.03 ± 1.26 (*P* = 0.001). A total of 13 (12.4%) patients had complications, with dry eyes (21.4%) being the most common manifestation. The complication rate was higher in female patients (16.4%) compared to male (5.3%, *P* = 0.048). Photo-refractive keratectomy had significantly higher complications rated (33.3%) compared to laser *in situ* keratomileusis (7.1%, *P* = 0.001).

**Conclusion:**

Refractive surgery significantly improved visual outcomes in hypermetropic patients. Dry eye was the predominant complication, with higher rates found in females and patients who underwent photo-refractive keratectomy. Studies with extended follow-up are needed to evaluate the long-term complications and sustained efficacy of refractive surgery in hypermetropic patients.

## Introduction

Refractive errors are one of the most common presenting complaints for ophthalmic patients. These conditions result from a congenital or acquired change in the axial length and/or refractive power of the lens in the eye, resulting in an image being created in front of or behind the retina in the eye^[^1^]^. Most prevalent among these refractive errors is myopia, followed by hypermetropia^[^1,2^]^. Hypermetropia, also called farsightedness, is a condition in which parallel light rays are concentrated behind the retina, and nearby objects appear blurry^[^2^]^.

Depending on the patient’s preference, the error can be corrected using spectacles or through surgical correction^[^3^]^. Currently, laser refractive surgery is a prevalent choice by the physician and patient due to its efficacy and prevention of risks associated with previous intraocular surgeries^[^3^]^. Over time, various laser refractive surgeries have been developed, with photo-refractive keratectomy (PRK) being the first surgery type, first performed three decades ago^[^4^]^. Currently, laser-assisted *in situ* keratomileusis (LASIK) is the preferred and most performed refractive correction technique^[^4^]^.

### Hyperopia correction techniques

The small incision lenticule extraction (SMILE) is considered to be the most recent method and is gaining popularity^[^3^]^. It involves using a femtosecond laser to create a corneal lenticule that is extracted whole through a small incision without the use of an excimer laser. It is reported to have the same effect of LASIK but with better outcomes^[^3^]^. However, the evidence is still limited and the long-term efficacy of SMILE for hyperopia correction^[^3^]^.

Another method to correct hyperopia is to place a thin segment of biological or synthetic tissue material into the corneal stroma, this is referred to a corneal inlay it can correct refractive error by increasing the central corneal curvature^[^3^]^.

Correction of hypermetropia with laser refractive surgery is considered more difficult and challenging, with fewer studies available to make evidence-based decisions.^[^3,5^]^ Complications of refractive surgeries vary from dryness and simple postoperative discomfort to postoperative ectasia, which is a visually threatening condition^[^4^]^. This study aims to assess the visual outcomes and complications in patients with hypermetropic eyes after refractive surgery and has been reported in line with the STROCSS guidelines^[^6^]^.

## Materials and methods

A retrospective cohort study was based on data collected from an Itqan Specialized Eye Center between January 2020 and the end of December 2022. Ethical approval was obtained from the appropriate institutional ethics committee. The study adheres to the Declaration of Helsinki and has been reported in line with the STROCSS guidelines^[^6^]^.

A total of 105 consecutive adult patients who underwent refractive surgery for hyperopia were included. The inclusion criteria are all patients who underwent refractive surgery. No sample size calculation or power analysis was performed before collecting the data, since the study relied on a consecutive sampling method based on available records within the mentioned period. The exclusion criteria comprised patients under the age of 18 years, pregnant women, and patients with local disease (e.g., keratoconus) that prevents them from undergoing refractive surgery.

Data collection form was used for collection over different variables including patients’ demographics: MRN, age, date of surgery, and sex; past medical history, type of refractive surgery, pre-operative assessment: best-corrected visual acuity (BCVA) in decimal form, central corneal thickness (CCT), and uncorrected visual acuity (UCVA) and short post-operative follow-up assessment (1st day, 1st week, 1st month): 1st day post-BCVA, 1 week UCVA, 1 month UCVA, and complications. VA was evaluated in decimal form for both near and far vision to detect post-operative over or under correction, if any. Literature search from 2018 until now, each study was reading carefully. Complications and adherence to post-intervention medication and instructions were verbally confirmed during follow-up visits by the attending physician. The primary outcome of this study is UCVA and the secondary outcome is postoperative complications and spherical equivalent (SE).

## Intervention details and considerations

All patients were evaluated at Itqan Specialized Eye Center by an ophthalmologist, optometrist, and optical technician during both the pre- and post-intervention phases. The procedures were performed in the operating theatres dedicated to refractive surgeries. The LASIK procedure is performed by creating a corneal flap using a femtosecond laser, followed by stromal ablation with an excimer laser. In contrast, PRK is performed by mechanical removal of the corneal epithelium and direct stromal ablation using an excimer laser. Both procedures were performed weekly by experienced refractive surgeons under topical anesthesia. Following surgery, all patients received topical moxifloxacin as a prophylactic antibiotic, Redfort (fluorometholone) steroid drops, and lubricating eye drops. Patients were instructed to avoid rubbing eyes and water exposure for at least 2 weeks. All patients presented with blurry vision and decreased near vision, majority of them were wearing either eyeglasses or contact lenses.

**HIGHLIGHTS**

- The study evaluated visual outcomes and complications in patients with hypermetropic eyes who underwent refractive surgery.
- Uncorrected visual acuity significantly improved after refractive surgery, from 0.62 ± 0.32 to 0.91 ± 0.27 in the right eye (*P* = 0.025) and from 0.62 ± 0.31 to 0.89 ± 0.27 in the left eye (*P* = 0.043).
- Photo-refractive keratectomy was linked to a significantly higher complication rate (33.3%) compared to laser *in situ* keratomileusis (7.1%, *P* = 0.001).
- Dry eye was the most common complication, especially among females who underwent photo-refractive keratectomy.
- Uncorrected visual acuity significantly improved after refractive surgery, from 0.62 ± 0.32 to 0.91 ± 0.27 in the right eye (*P* = 0.025) and from 0.62 ± 0.31 to 0.89 ± 0.27 in the left eye (*P* = 0.043).
- Photo-refractive keratectomy was associated with a significantly higher complication rate (33.3%) compared to laser *in situ* keratomileusis (7.1%, *P* = 0.001).
- Dry eye was the most frequent complication, particularly among females who underwent photo-refractive keratectomy.

## Data analysis

The data were collected, reviewed, and then transferred to Statistical Package for the Social Sciences (SPSS) version 21, a product of IBM Company. Categorical variables were described using frequency and percentage. All ophthalmic measures in both eyes during different assessment phases were displayed using interquartile range with median and mean with standard deviation according to data distribution. Changes across visits were assessed using repeated-measures ANOVA. post hoc pairwise comparisons were performed with Bonferroni adjustment. The graphical presentations for displaying measurement changes were used. The patients were divided into two subgroups based on the type of surgical procedure to compare the study outcomes. Cross-tabulation was used for subgroup comparisons, and statistical significance was assessed using Pearson’s chi-square test or Fisher’s exact test for small sample distributions. Two-tailed *P* value of less than or equal to 0.05 was considered significant for all statistical methods.

## Results

A total of 105 eligible patients were included. Patients’ ages range from 19 to 61 years with a mean age of 38.1 ± 12.0 years. The majority (63.8%) of the patients were females. As for surgery undergone, LASIK was the most common surgery with 84 (80%) cases and followed by PRK which was done on 21 (20%) patients (Table 1). the central corneal thickness in both eyes among patients with hypermetropic eye undergone refractive surgery. CCT in the right eye ranged from 490 to 654 with a mean value of 559 ± 37 compared to 497 to 650 in the left eye with a mean value of 560 ± 37 with no statistically significant difference (*P* = 0.316).

**Table 1:** Demographic and surgery data of study patients with hypermetropic eye undergone refractive surgery

Demographics	No	%
Age in years		
<30	34	32.4%
30–39	22	21.0%
40+	49	46.7%
Mean ± SD	38.1 ± 12.0	
Gender		
Male	38	36.2%
Female	67	63.8%
Types of surgery		
LASIK	84	80.0%
PRK	21	20.0%

**Figure 1.**
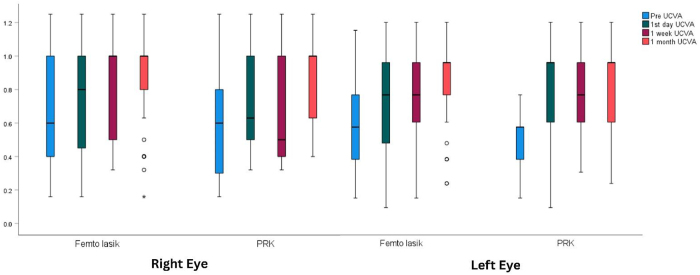
Uncorrected visual acuity (UCVA) in the right and left eyes before and after refractive surgery.

Table 2 represents the UCVA in both eyes before and after refractive surgery. It showed a statistically significant increase in 1-month post-operative UCVA with the right eye increasing from 0.62 ± 0.32 to 0.91 ± 0.27 and the left eye rising from 0.62 ± 0.31 to 0.89 ± 0.27 (*P* = 0.025 and 0.043, respectively). As for UCVA changes by surgery type, both eyes showed a significant increase in both types of surgery, mainly in LASIK surgery (Fig. 1).

**Table 2:** Uncorrected visual acuity (UCVA) in both eyes before and after refractive surgery

Phase of assessment	Right eye		Left eye		*P* value
	Mean	SD	Mean	SD	
Pre-operative	0.62	0.32	0.62	0.31	0.954
1st day post-operative	0.76	0.30	0.73	0.28	0.841
1-week post-operative	0.79	0.29	0.82	0.28	0.395
1-month post-operative	0.91	0.27	0.89	0.27	0.847
*P* value	0.025*		0.043*		

P: Adjusted P value of repeated measures ANOVA

^*^ P < 0.05 (significant)

**Figure 2.**
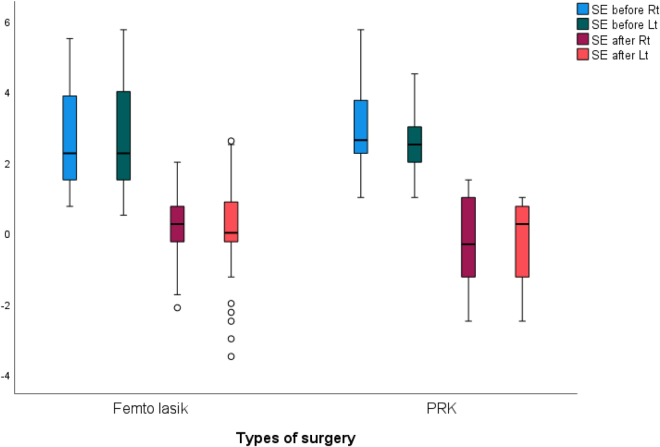
Spherical equivalent (SE) in study patients before and after refractive surgery for both eyes.

Table 3 shows spherical equivalent (SE) among study patients before and after surgery. In the right eye, SE significantly changed from 2.47 ± 1.24 to 0.19 ± 0.69 (*P* = 0.001). Also, the left eye showed a significant average change from 2.64 ± 0.173 to −0.03 ± 1.26 (*P* = 0.001). Similar reduction in SE was reported in both eyes by the surgery type (Fig. 2).

**Table 3:** Spherical equivalent among study patients before and after surgery

SE	Minimum	Maximum	Mean	SD	*P* value
Right eye					0.001*
*Before surgery*	0.75	5.75	2.47	1.24	
*After surgery*	−2.50	2.00	0.19	0.96	
Left eye					0.001*
*Before surgery*	0.50	5.75	2.64	1.73	
*After surgery*	−3.50	2.60	−0.03	1.26	

P: Wilcoxon test

^*^ P < 0.05 (significant)

Regarding the complications reported among study patients with hypermetropic eye undergone refractive surgery. A total of 13 (12.4%) of the study cases had complications which were dry eyes (21.4%), mild allergic conjunctivitis (14.3%), pain (14.3%), and other complications each with 1 case. All complications noted for both interventions are considered grade I based on the Clavien–Dindo Classification and only required an increased frequency of lubricating eye drops or the prescription of night gel.

Table 4 the factors associated with complications among patients with hypermetropic eye undergone refractive surgery. A total of 16.4% of female patients had complications versus 5.3% of male with recorded statistical significance (*P* = 0.048). Also, 33.3% of patients undergone PRK had complications compared to 7.1% of those with LASIK (*P* = 0.001).

**Table 4:** Factors associated with complications among patients with hypermetropic eyes undergoing refractive surgery

Factors	Complications				*P* value
	Yes		No		
	No	%	No	%	
Age in years					0.249
<30	3	8.8%	31	91.2%	
30–39	5	22.7%	17	77.3%	
40+	5	10.2%	44	89.8%	
Gender					0.048*^
Male	2	5.3%	36	94.7%	
Female	11	16.4%	56	83.6%	
Types of surgery					0.001*^
Lasik	6	7.1%	78	92.9%	
PRK	7	33.3%	14	66.7%	

P: Pearson X^2^ test; ^: Exact probability test

^*^ P < 0.05 (significant)

## Discussion

Refractive errors are the most common complaint among ophthalmic patients, affecting patients of all age groups and are recognized as a public health concern^[^7^]^. Hypermetropia, also called far-sightedness or long-sightedness, is when due to an error in lens or axial length the unaccommodating eye is unable to focus the parallel light rays on the fovea^[^8^]^. Instead, the rays are focused behind the retina^[^8^]^. Surgical correction of hypermetropia performed through laser refractive surgeries offers optimal efficacy with the prevention of the risks associated with intraocular surgeries. While multiple types of laser surgeries are now available, PRK and LASIK are the frequently performed methods to correct hypermetropia, with LASIK being more commonly used^[^3^]^. This study evaluates the visual outcomes in patients with hypermetropic eyes after refractive surgery.

CCT is an important preoperative factor that should be measured before performing excimer laser refractive surgery^[^9^]^. In our study we measured central corneal thickness in both eyes preoperatively, CCT in the right eye ranged from 490 to 654 with a mean value of 559 ± 37 compared to 497-650 in the left eye with a mean value of 560 ± 37 with no statistically significant difference (*P* = 0.316). This study found UCVA improves following surgery in both eyes, in the right eye, it increased from 0.62 ± 0.32 to 0.91 ± 0.27, and in the left eye, it increased from 0.62 ± 0.31 to 0.89 ± 0.27 (*P* = 0.025 and 0.043, respectively). After undergoing both types of surgery, primarily LASIK, both eyes demonstrated a notable rise. According to a prior study in Germany keratometric alterations following corneal laser refractive surgery occur up to 6 months after LASIK and for at least 6 months following PRK^[^10^]^.

Stein *et al*’s study looked at the epithelium healing time, complication rate, postoperative SE, UCVA, and BSCVA^[^9^]^. It was shown that eyes that had LASIK had less discomfort, faster visual recovery, and less hyperopic regression, even if similar SE was eventually obtained.^[^8–10^]^ According to a study from Barcelona, all the procedures used in the study had a statistically significant association between the SE in absolute value and dismal UDVA, with the correlation reaching higher values in the FS-LASIK and LASIK techniques, at 0.774 and 0.706, respectively^[^11^]^. These studies support our findings that LASIK surgery has better outcomes in uncorrected visual acuity.

Another study discussed the evolution of flap thickness, optical density, and visual acuity following femtosecond laser *in situ* keratomileusis. In terms of obtaining LASIK flaps, the femtosecond laser study seems to be a reliable, safe, and consistent platform. An efficacy of 0.98 ± 0.1, safety of 0.98 ± 0.1, and reliable predictability with 100% of eyes within ± 0.5 D of emmetropia were all well achieved with the femtosecond laser at three months after surgery^[^12^]^.

Results of our study show that SE significantly changed in the right eye from 2.47 ± 1.24 to 0.19 ± 0.69 (*P* = 0.001). Also, the left eye showed a significant average change from 2.64 ± 0.173 to −0.03 ± 1.26 (*P* = 0.001). A similar reduction in SE was reported in both eyes by the surgery type. In a previous study, the efficacy and safety of PRK and LASIK in the treatment of hyperopia were compared, and it was found that there was a minimal change in the mean SE of the LASIK group^[^13^]^. The study found a mean regression of +0.016 D per month and mean SE at 12 months of +0.37 D^[^13^]^. While the same study found that the PRK cohort has a significant myopic correction, with mean SE −0.82 D (*P* < 0.001) at 1 month^[^13^]^. This difference in mean SE was not significant at 3 and 6 months follow-up between PRK and LASIK^[^13^]^. In contrast, PRK showed a regression rate of +0.20 D per month, at 1 and 6 months, while LASIK had rate of +0.02 D per month^[^13^]^. Another study conducted in Saudi Arabia also manifested SE regression 1 year after LASIK however, there findings were not statistically significant (*P* = 0.46)^[^14^]^. A study conducted in 2019, aimed to evaluate the safety and efficacy of LASIK for high hypermetropia correction at 1 week till 1 year found significant SE reduction at from 4.69 ± 1.20D (+3.75 to +7.50D) to 0.58 ± 0.56D (+0.25 to +0.88D) (*P* < 0.0005)^[^15^]^.

Complications from refractive surgery were identified in our study among patients with hypermetropic eyes and were observed in 13 cases, accounting for 12.4% of the total cases. The most commonly reported complication was dry eye, occurring in 21.4% of the cases with complications. When comparing these findings to previous studies, a notably higher complication rate was observed in one earlier study, where 20 eyes (39.2%) experienced postoperative complications^[^15–17^]^. Among them, long-lasting dry eye was reported in 3 eyes (5.88%), indicating a lower relative incidence of dry eye compared to our study^[^16^]^. Similarly, another study documented prolonged dry eye symptoms in nine patients (18 eyes), accounting for 6.7% of eyes even three months postoperatively^[^15^]^. The evidence revealed that more than 90% of cases of the various postoperative problems that might arise with LASIK, like dry eye, are observed in the early postoperative phase. It has been found that 5–51% of cases had residual refractive error after LASIK. Other flap related complications may include striae and dislocation of the flaps. These eyes can be prevented from losing their eyesight with early detection and prompt treatment^[^17^]^.

This study found that certain patients have a statistically significant association with post-operative complications. Complications were more frequent among female patients (16.4%), while only 5.3% of male patients developed complications (*P* = 0.048). Furthermore, patients undergoing PRK were more likely to develop complications than those undergoing LASIK (33.3% vs 7.1%, *P* = 0.001, respectively). A different study reported PRK compared to LASIK, had higher postoperative astigmatism^[^18^]^. The therapeutic outcomes of hyperopic PRK may be enhanced by larger optical zones and newly developed ablation profiles that result in a smoother ablation surface^[^18,19^]^. According to the results of another study conducted in Croatia, trefoil after PRK is worse than LASIK. After LASIK, corneal changes are more predictable^[^19^]^. Our findings are in accordance with their conclusion.

When comparing the side effect of both procedures, PRK offers the highest biostability and the quickest restoration of normal corneal sensitivity; however, it involves the longest recovery time, worst postoperative discomfort, and a higher risk of corneal haze^[^20,21^]^. In contrast, LASIK provides the fastest visual recovery but has a potential risk of lifetime flap displacement^[^20,21^]^. Additional complications of PRK may include temporary blurred vision, photophobia, and, in rare cases, infections^[^20^]^. In contrast, the corneal flap done in LASIK can cause flap displacement, striae, or epithelial ingrowth^[^21^]^.

In contrast to previous studies, the strength of our study is that we compared the outcomes and complications of two types of refractive surgery, LASIK and PRK. Most hypermetropia patients did not prefer refractive surgery but tried to accommodate with their near vision by binding things to distance. Another limitation is that our study population was selected from a single clinical site, so a limited number of patients were analyzed. Lastly, the duration was during and directly after coronavirus 2019 (COVID-19) restrictions and lockdown.

## Conclusion

Refractive surgeries can significantly improve visual outcomes in hypermetropic patients, with the LASIK technique showing superior results compared to PRK in terms of UCVA improvement. The most common reported complication was dry eyes, and the highest incidence of complications was identified in female patients and those undergoing PRK. Studies with extended follow-up are needed to evaluate the long-term complications and sustained efficacy of refractive surgery in hypermetropic patients.

## Data Availability

The data are available upon reasonable request.

## Research registration unique identifying number (UIN)

researchregistry11078.

## Compliance

The study adheres to STROCSS criteria (Strengthening the Reporting of Cohort Studies in Surgery) and ICMJE recommendations.
